# An exploratory investigation of the influence of publication on translational medicine research

**DOI:** 10.1186/1479-5876-8-62

**Published:** 2010-06-23

**Authors:** Lisa J Douet, Danielle Preedy, Vaughan Thomas, Ian A Cree

**Affiliations:** 1 NIHR Evaluation, Trials and Studies Coordinating Centre, University of Southampton, SO16 7NS, UK

## Abstract

**Background:**

Changes in clinical practice are brought about by the weight of clinical evidence for and against an intervention. Clinical evidence of efficacy relies on the dissemination of research results, usually by publication in medical journals which is often seen as a pre-requisite for progression of an intervention through further clinical trials or implementation studies.

**How far has research progressed along the translational pathway?:**

We undertook an exploratory exercise to determine where basic and translational medical research is currently published. Original research articles (329 in total) published in high impact general and specialist medical journals were classified into different stages of research within the translational medicine pathway.

**Where is translational research published?:**

The general medical journals had the broadest spread of published research over the translational pathway. The specialist journals tended to be positioned to disseminate the research findings of early stage translational research from basic science results through to early stages of clinical testing.

**Conclusion:**

It is not possible for one journal to satisfy all the needs of the reader and the author along the translational medicine pathway. For an intervention to progress along the translational pathway background information should be readily accessible in the article. This pathway is currently being actively managed by the funding agencies but the next challenge is to ensure the pathway operates efficiently and does not allow promising innovations to languish and to provide a smoother transition for interventions to reach the clinic in a quicker timescale. It is clear that the dissemination of results in the right place at the right time is crucial to the transition of an intervention from the laboratory to clinical practice.

## Introduction

**Figure 1:**
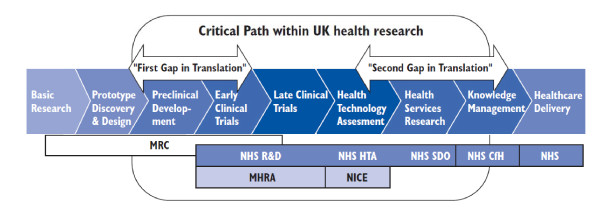
**Cooksey report research pathway**. The Cooksey report identified two gaps in the translation of biomedical science to healthcare: The first gap arises in the translation of basic and clinical research into ideas and products; the second gap relates to introducing those ideas and products into clinical practice[6].

The status of translational research (defined as the progression of scientific advance from inception to clinical practice) has drawn increasing attention as it is seen to be essential to the development of new treatments from advances in biomedical science [1-5]. In 2006, the NIH introduced the Bench to Bedside awards, with the aim of encouraging collaboration between clinicians and basic scientists across institutes. In the same year the Cooksey review [6] was commissioned to undertake an independent review for the public funding of health research in the UK. The report identified two key gaps in the transitional medicine pathway, described as the early phase and the late phase gaps (Figure 1). These gaps limited the progress of new interventions through the translational pathway.

**Figure 2:**
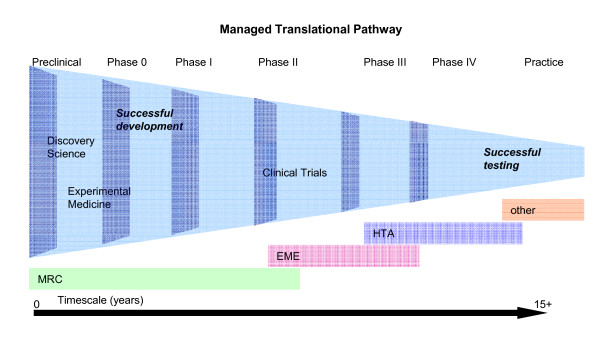
**The managed translational pathway**. This is the managed translational pathway between the MRC and NIHR. The overlap between the stages is deliberate - it is quite possible that some studies or trials could be funded by more than one body; this overlap is deliberate to ensure that studies do not fall between gaps. Successful publication and a managed pathway should led to faster translation through this pathway, which currently van take at least 15 years from discovery to changes in clinical practice.

Within the UK, the Cooksey report resulted in the reorganisation of research funding and the creation of several new funding programmes, with the aim of producing a managed translational pathway (Figure 2) which would not lose good ideas in the gaps which previously existed between programmes. In terms of government funding for medical research within the UK, the Medical Research Council (MRC) retained funding for basic research and early clinical studies [7]. The Efficacy and Mechanism Evaluation (EME) programme [8] was created to fund efficacy studies and the established NIHR Health Technology Assessment (HTA) programme [9] continues to fund effectiveness research.

The aim of the translational pathway is to ensure that clinical studies for new treatments or diagnostics at all stages of development or evaluation have the potential to gain funding. For the purpose of this article the translational pathway has been divided into four categories of research; early, efficacy, effectiveness and other. The early research category covers research and development for basic discoveries and for new therapeutic/diagnostic/device/public health intervention (or a new indication for an existing intervention) from basic science through to early phase clinical studies with the aim of establishing proof of concept in humans. The efficacy category includes all research which aims to establish definitive proof of efficacy for an intervention (where proof of concept has already been achieved), to the point where, in the example of a drug, marketing authorisation could be sought or widespread use in healthcare supported. The effectiveness category includes all research which aims to investigate effectiveness, costs, and the broader impact of health technologies for those who use, manage and provide care in the health service. The other research category encompasses all research which is not covered by the previous three categories; research that falls into this category generally sits further along the translational pathway and includes service delivery, health service, global health and public health research as well as epidemiology and modelling. The challenge is to ensure that the benefits of translational research are delivered as changes in clinical practice.

Changes in clinical practice are brought about by the weight of clinical evidence for and against an intervention. This involves assessment of the quality of the evidence, the risks and benefits of treatments, and the impact of implementation on clinical care pathways by practitioners and those determining healthcare policy. Clinical evidence of efficacy relies on the dissemination of research results, usually by publication in medical journals which in academic models is often seen as a pre-requisite for progression of an intervention through further clinical trials or implementation studies. Therefore, publication of translational research is a critical step - how and where, results are published will often have an impact on further progression through the pathway and can inform research managers where to target their resources. A recent review identified 101 articles between 1979-1983 in 6 top basic science journals that had apparent promise for development as a major clinical application. But over twenty years later, only five of these promising advances were in licensed clinical use and only one of them had had a major impact on current medical practice. Three quarters of the promised interventions resulting from these basic science papers had not yet been tested in a randomised trial. The article reported that the strongest predictor of moving to randomised experimentation was industry involvement in the original basic science publication [10]. Clearly, the publicly funded research system has not been working as effectively as one might wish.

## How far has research progressed along the translational pathway?

Publication is seen as a major output for research, though clinical impact is its goal, as well as having financial implications for the research groups involved through research assessment exercises that often use publications as a surrogate. It is certainly true that the readership of a journal varies, and it is possible that in some cases, publication in specialty rather than general journals may be more appropriate and influential in the progression of an intervention. To investigate if this is possible we undertook an exploratory exercise to determine where basic and translational medical research is currently published.

Two authors (LD and IC) independently classified original research articles (329 in total) published in the high impact general medicine journals Nature Medicine, New England Journal of medicine (NEJM) and The Lancet from July-December 2008 and one month (October 2008 was chosen as it was in the middle of the time frame studied) of seven high impact speciality journals; American Journal Respiratory Critical Care Medicine (AJRCCM), Circulation, Gut, Journal Clinical Oncology, Journal of Orthopaedic Research, Lancet Neurology and finally Ophthalmology. The articles were classified into one of four phases discussed in the introduction; early research, efficacy research, effectiveness research, and other research. In some of the papers reviewed the information needed by the reader to determine where the research sat along the translational pathway was not always readily available within the article. Articles over which there was disagreement over the classification (45 articles, 13.6%) were independently classified by a further two authors (VT and DP). Any remaining disagreements over articles were resolved by discussion between all four authors.

## Where is translational research published?

**Figure 3:**
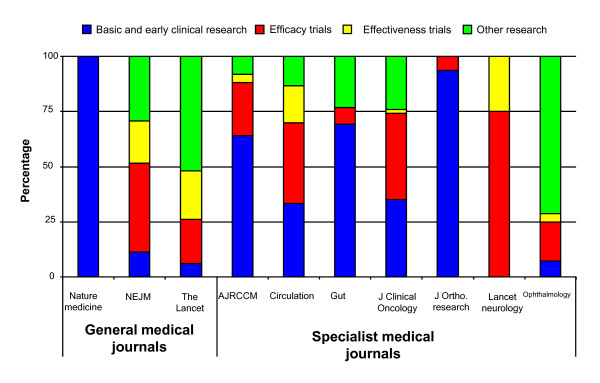
**Primary research article classification**: Published primary research articles categorised by research phase (basic and early clinical research, efficacy research, effectiveness research and other research) as a percentage of the total number of articles for that journal during the period July-December 2008 for the general medical journals and for October 2008 for the specialist medical journals.

From our exploratory study it has been possible to detect some early trends in the research published by the selected journals. The general medical journals, The Lancet and NEJM had the broadest spread of published research over the translational pathway. In the time frame investigated the NEJM published more efficacy research (40% of articles) than early research or effectiveness research, which combined constituted only 30% of the articles. Whereas, all stages of translational medicine were more equally represented in The Lancet. In the period studied both efficacy and effectiveness research was well represented in this journal, with each of these categories contributing 20% to the total number articles published. The 'other research' category was well represented by these two journals and although not clearly demonstrated by figure 3, these were the only two journals of those selected to provide a forum for global health research. Both NEJM and the Lancet had the smallest proportion of original articles devoted to basic science, which represented 11% and 6% respectively, whereas, Nature Medicine seemed to be clearly positioned at the basic science and early clinical studies end of the translational pathway, with 100% of its articles falling into this category.

From the limited selection of specialist journals analysed it appeared to be clear that they are positioned to disseminate the research findings of early stage translational research from the basic science results through to the early stages of clinical testing; all journals, with the exception of ophthalmology, consist of at least 50% early and efficacy research. Effectiveness research is much less prevalent in the selected specialist journals with only Circulation and Lancet Neurology publishing a noteworthy number of these trials at 17% and 25% respectively (figure 3).

Again the other research section is not further analysed here, but Gut, Circulation, Ophthalmology and Journal of Clinical Oncology publish a substantial amount of research in the public health and service delivery aspects of medicine (figure 3). Epidemiology and modelling contributes a large proportion of the articles published in Journal of Clinical Oncology and Ophthalmology.

The specialist journals are also limited by how far the interventions for their speciality are along the translational pathway, for example orthopaedic research involves a lot of basic research which is only just starting to be translated into clinical studies. If this analytical approach to studying publications were expanded it seems likely that it could be exploited by research commissioners to ensure promising treatments are actively translated into clinical practice.

## Conclusions

It is obviously not possible for one journal to satisfy all the needs of the reader and the author along the translational medicine pathway. Even in this small sample of journals the positioning of the journal in the translational pathway is likely to affect the chance of an article falling within the first gap in translation being published in the journal of choice. The general medical journals The Lancet and the NEJM publish a wide range of research that reflects the wide ranging readership of the general medical journals, though the recent study in Science [10] suggests that this may not often translate into practical medical advances. There is no doubt that the majority of early translational work is published in the specialist journals. This article has focused on a select few of the high impact specialist journals, but there are many more journals with lower impact factors that have a smaller (and perhaps more sub-specialist) audience than the largest specialist journals. It is possible that key research findings along this pathway may be missed. Important and high quality work may be lost in these journals if it is not of more general interest at that moment and is not pursued by the research group concerned. This is now obviated, to some extent, by the widespread use of search engines, such as PubMed.

Some of the articles were difficult to categorise, even with four independent opinions, as the background information on the research was not always available, without searching the references. This has important implications for the development of the clinical evidence base as some of the research may be overlooked as it is not clear where the intervention is along the translational pathway. It is debatable whether any intervention pursues a linear progression along this artificial pathway: as often multiple advances, both scientific and technical, lead to a change in clinical practice.

However, in our view, it is essential that the managed translational pathway should prevent the potential loss of valuable information by actively managing it through the different stages involved. This pathway is currently being actively managed by the funding agencies. The focus is now to ensure that the pathway operates efficiently and does not allow promising innovations to languish without a funder, providing a smoother transition for interventions to reach the clinic in a quicker timescale. The challenge is to ensure that the benefits of translational research are delivered as changes in clinical practice. From this exploratory study, it appears that the dissemination of results in the right place at the right time can be critical to the transition of an intervention from the laboratory to clinical practice and the medical journals have an important role in supporting this process. Further work will needed to draw firm conclusions, but we hope that these results will provoke thought and discussion as to how publication could better influence translation of research into clinical practice.

### Summary points

1. Where an article is published mirrors the progression of an intervention along the translational medicine pathway.

2. The journals chosen appeared to publish work which is at particular stages along the translational pathway.

3. To evaluate the progress of an intervention along the translational pathway it is helpful if previous work is described to clearly demonstrate where the intervention is within the pathway.

4. The medical journals have a key role alongside the funders of the managed translational pathway to ensure interventions move from basic science to clinical practice quickly and smoothly.

## Competing interests

All authors declare that the answer to the questions on your competing interest form are all No and therefore have nothing to declare.

## Funding statement

This research was carried out by the NIHR Evaluations Trials and Studies Coordinating Centre. No additional funding was sought for this research.

## Ethical approval

Ethical approval was not required for this research.

## Contribution of author

LD and IC were involved in all stages of this study; DP and VT assisted with classification of articles. All authors critically reviewed and agreed the final draft. LD and IC are the guarantors.
